# Pharmacoeconomic analysis of antibiotic therapy in maxillofacial surgery

**DOI:** 10.1038/bdjopen.2017.2

**Published:** 2017-02-10

**Authors:** Bogusława Orzechowska-Wylęgała, Adam Wylęgała, Michał Buliński, Iwona Niedzielska, Andrzej Madej

**Affiliations:** 1 Department of Cranio-Maxillofacial Surgery, Medical University of Silesia, Katowice, Poland; 2 Department of Ophthalmology Santa Barbara Hospital, Sosnowiec, Poland; 3 Department of Internal Diseases and Subdepartament of Cardiac and Diabetology Unit, The Boni Fratres Catoviensis Hospital, Katowice, Poland

## Abstract

**Objectives/Aims:**

The aim of this study was to investigate the microbial cultures collected in the years 2013–2014 at the craniomaxillofacial department and outpatient clinic to analyse optimisation of the treatment cost of the bacterial infections and present the results.

**Design and setting:**

We analysed 485 cultures from 263 patients, of which 77.28% consisted of Gram-positive bacteria. On the basis of the antibiotic efficacy, antibiotic price and the cost of entire treatment during hospitalisation, the most useful antimicrobial agents for the most common pathogens were selected.

**Results:**

The most frequently collected material was pus. The most common pathogens were found to be the *Staphylococcus epidermidis* (18%), *Streptococcus mitis* and *Str. oralis* (14%) and *S. aureus* (6.5%).

**Discussion:**

The most frequently isolated bacteria in other studies were the *Streptococcus* strain. Other authors showed that ceftriaxone is the most cost efficient agent. The use of postoperative antibiotic prophylaxis remains controversial.

**Conclusion:**

The results of this study suggest that the most useful antibiotics for therapy, from the perspective of the cost minimisation, were gentamycin, trimethoprim with sulfamethoxazole and vancomycin.

## Introduction

Recommendations on the administration of antibiotics as perioperative prevention has been elaborated for the Ministry of Health, the study do not provide a detailed review of maxillofacial surgical procedures, focusing primarily on ear nose throat (ENT) surgery. There are no pharmacoeconomic analyses, which look into the medications used in maxillofacial surgery.^1^

The growing antibiotic resistance forces us to elaborate well-grounded and economically viable criteria pertaining to the use of antimicrobials. Antibiotics are often prescribed in a manner that is schematic and unreasonable (on patient’s request), particularly, in viral infections or fever of unknown aetiology, which results in greater number of antibiotic-resistant bacterial strains and multidrug-resistant organisms.^2^ Multiple drug resistance is a significant problem, where a given microorganism becomes resistant to several groups of antibiotics.^3^ Apart from the rational use of antibiotics, it is also of key importance to adhere to an appropriate dosing schedule. In order for the treatment to be efficacious, one has to determine the aetiology involved and examine the antibiotic sensitivity of the microorganism in question.^4^

The aim of the study was to present the results of cost minimisation analysis with reference to bacterial infections based on microbial cultures collected in the years 2013–2014 at the craniomaxillofacial department and outpatient clinic.

## Materials and methods

In the period of 1 January 2013–31 December 2014, a total number of 485 bacterial and fungal strains were cultured from 263 patients (112 females and 151 males; Table 1) treated at the Department of Cranio-Maxillofacial surgery and outpatient maxillofacial clinic. The patients were aged 10–79 (mean age: 41.3) years. The material collected for studies was primarily pus from submandibular and submental abscesses (55%), maxillary sinus swabs from sinusitis (12.5%), cutaneous fistula from submandibular regions (10%), and wound swabs from various facial regions (6%) as well as bone swabs from mandibule (4%). All the microbiological samples were analysed in the bacteriological unit of the Central Laboratory at the SPSK-M. First, the Gram-stained bacteriological preparations were made. Fungi of the genus Candida were identified by means of Candida ID bioMerieux chromogenic plates (BIOMERIEUX, Marcy-l'Etoile, France) and the Auxacolor 2 test by Bio-Rad Laboratories Inc. (Hercules, CA, USA). Antibiograms were prepared using VITEK 2 compact bioMerieux analyser (BIOMERIEUX). In the automatic method, antibiograms were made with a Vitek 2 compact analyser using AST-P 534 and AST-P-533 cards for other streptococci, AST-P 536 for staphylococci, and AST-N 019 AST-N022 for Gram-negative bacteria. The cards, AST-P-586, AST-P-576 and ST01, were used for streptococci, AST-P-580 for staphylococci, whereas AST-N84, AST-N259, AST-N93 and AST-N260 were used for Gram-negative bacteria. Antibiogram interpretation concerning the disk method is as following: susceptible, semisusceptible and of resistance. The antibiograms performed on cards were following: susceptible, semisusceptible and resistance; and it is defined as minimum inhibitory concentration, the lowest antibiotic concentration that can inhibit the growth of a given microorganism.

Gram-positive and Gram-negative bacteria were compared for their sensitivity to nine antimicrobial agents. The results achieved were subjected to the Fisher test statistical analysis, with *P*<0.05. The one-way analysis of variance with Dunnett’s *post hoc* test was carried out with the use of Pearson’s *χ*^2^ test. GraphPad Prism version 5.00 for Windows, GraphPad Software, San Diego, CA, USA, was used for the purpose.^5^

A series of calculations were performed to determine the antibiotics that had been the most beneficial in empirical treatment of conditions caused by the most common pathogens. Antibiotic efficacy, antibiotic price and the cost of entire treatment during hospitalisation were considered. The following mathematical model was elaborated for calculations.

$$7(\prod_{k=1}^{s}p_{1}+\prod_{k=1}^{r}p_{2})+3(\prod_{k=1}^{r}p_{1}+350r)$$

*s*—percentage of bacteria sensitive to the antibiotic, *p*_1_—price of empirical antibiotic therapy, *p*_2—_price of targeted antibiotic therapy, *r*—percentage of bacteria resistant to the antibiotic and 350 PLN—gross cost of patient day (when in hospital).

### Criteria for the cost minimisation analysis

1. Cost of empirical antibiotic therapy involving a sensitive bacterial strain.
2. Cost of 3-day-long empirical antibiotic therapy involving a resistant bacterial strain.
3. Cost of vancomycin-targeted treatment in drug-resistant patients.
4. Total cost of 3-day-long empirical therapy in drug-resistant patients, followed by the 7-day-long targeted treatment.
5. Total treatment costs for drug-sensitive and drug-resistant patients.
6. The result that was extrapolated for a single patient.

The cost minimisation analysis was carried out for the prevalent bacteria isolated in 2013 and 2014 at the Department of Cranio-Maxillofacial Surgery at the maxillofacial outpatient clinic. The dominant strains were *S. epidermidis*, *Streptococcus mitis* and *Str. oralis* as well as *S. aureus*. Better insight into the issue of drug resistance will allow us to estimate the number of patients in whose case a change of antibiotic will be necessary.

## Results

In 2013–2014 (Table 2), the most prevalent strain among the isolated Gram-positive bacteria was *Staphylococcus* *epidermidis*, totalling 48 strains (19.1%) in 2013 and 40 strains (17.2%) in 2014. *Str. mitis* and *Str. oralis* were second, constituting 37 strains (14.7%) of the cultured strains in 2013 and 30 strains (12.9%) in 2014 (*P*=0.24264).

Regarding the Gram-negative bacteria in the years 2013–2014, 19 were *Klebsiella pneumoniae* strains (3.9%), 17 *Escherichia coli* strains (3.5%) and 14 were *Haemophilus* strains (2.9%). In the final 2 years, there was also a decrease in the number of the *Enterobacteriaceae* rods cultured, totalling seven strains (2.8%) in 2013 and it was five strains (2.15%) in 2014 (*P*=0.24264).

In our studies, gentamycin showed statistically significant stronger effect than oxacillin (odds ratio (OR)=3.05, *P*=0.003), tobramycin (OR=4.753, *P*<0.0001) and penicillin (OR=27.41, *P*<0.0001). In contrast, clindamycin, tetracycline and erythromycin are weaker than gentamycin by 0.2694 (*P*<0.0001), 0.1734 (*P*<0.0001) and 0.1638 (*P*<0.0001) respectively.

**Figure 1:**
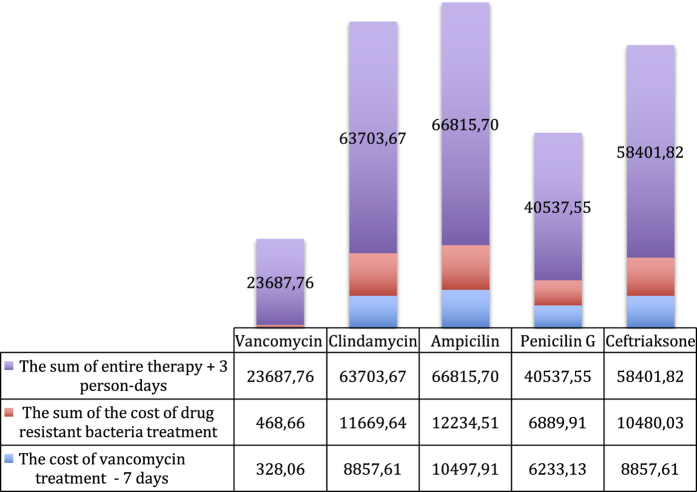
The graph presents the costs of treatment of the infection caused by *Streptococcus mitis* and *Str. oralis* with particular antibiotics.

**Figure 2:**
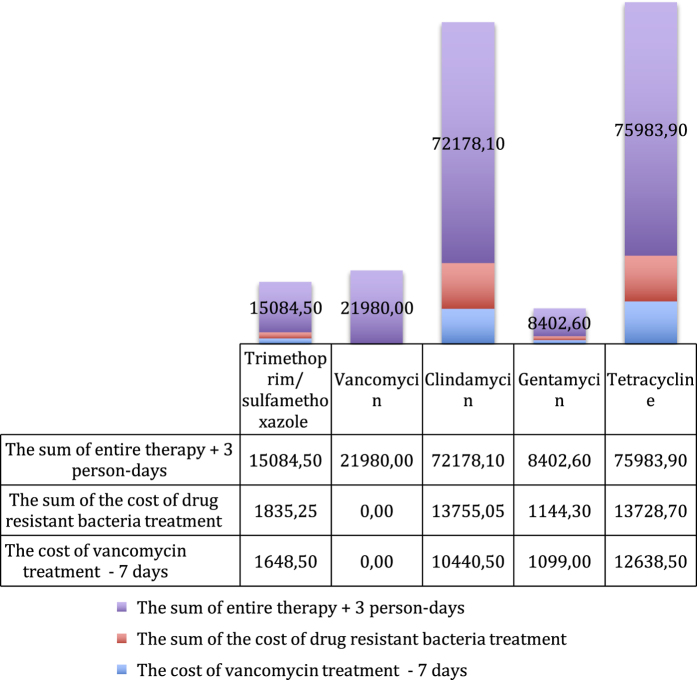
The graph presents the costs of treatment of the infection of *Staphylococcus epidermidis* with particular antibiotics.

**Figure 3:**
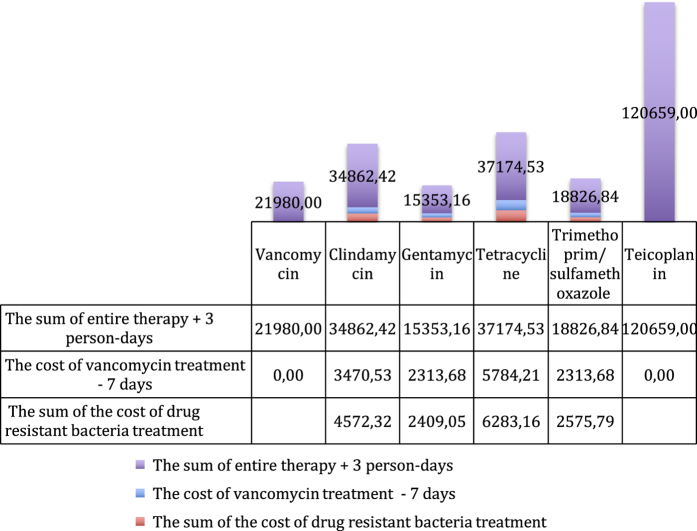
The graph presents the costs of treatment of infection of *Staphylococcus aureus* with particular antibiotics.

From the perspective of cost minimisation, the most advantageous antibacterial drug, due to its high efficiency towards *Str. mitis* and *Str. oralis*, turns out to be vancomycin (Figure 1), in which case the total costs of the therapy of 100 patients amount to 21,980 PLN, compared to the 58,090 PLN for cetrifaxone. This is because resistant bacteria will have to be treated with vancomycin. In contrast, gentamycin turns out to be the most advantageous against *S. epidermidis* (Figure 2), where the cost of the therapy is 8,402.6 PLN, whereas for trimethoprim/sulfamethoxazole, it is 15,084.5 PLN. The cost of treatment of *S. aureus* infections is the most beneficial with gentamycin (Figure 3), as it amounts to 15,353.16 PLN; and for trimethoprim/sulfamethoxazole treatment, it is 18,826.84 PLN.

## Discussion

Yuvaraj^6^ showed the domination of aerobic bacteria in a group of 88 patients with maxillofacial infections, where the most frequently isolated bacteria were *Streptococcus*. These results are similar to those obtained in our study. However, the researchers obtained much higher percentage of penicillin sensitivity in their study, equalling to 81%, in comparison to the 50.6% sensitivity in the group examined in our study. According to Molander *et al.*,^7^ *Enterococcus* has been the most frequently isolated strain in 100 cases of root-filled teeth with apical periodontitis. The researchers have showed very small proportions of obligate anaerobic Gram-negative isolates. This was also presented in our study.

Similar results were obtained by Ru *et al.*,^8^ who have also shown that the most frequently isolated bacteria were the *Streptococcus* strain. *S. epidermidis* was detected in 19% patients. This strain is a commensal bacterium colonising skin and mucous. It might also be due to swab contamination; however, *S. epidermidis* produces many virulence factors and forms biofilm, and was isolated in sinusitis and is now considered as one of the major source of nosocomial infections.^9–12^ Furthermore, *S. epidermidis* are the important source of infections of implants.^13,14^ Zix *et al.* in Switzerland carried out randomised, double-blinded placebo-controlled study concerning the application of perioperative therapy with antibiotics in 62 patients with orbit fractures and in 94 patients with maxillary and orbital–maxillary–zygomatic fractures. The patients were given amoxicillin with clavulanic acid for the period of 1–5 days before the surgery. It was shown that 1-day prophylaxis is sufficient in preventing inflammatory complications compared to the 5-day prophylaxis in all types of fractures.^15^ Similar observations were made by Andreasen *et al.*^16^ in Denmark, who have shown that a single dose or 1-day administration of antibiotic is sufficient to prevent infection in the surgical treatment of mandibular fractures and that it performed better than a 7-day therapy. This is a very important proof for the lack of necessity to prolong treatment with antibiotics, and thus to culture resistant strains. On the other hand, Miles *et al.*^17^ in the United States have not observed any statistically significant difference in a group of 181 patients treated surgically for mandibular fractures, irrespective of the fact whether they were given antibiotic postoperatively or not. The observations from our clinic allow us to avoid antibiotic therapy in the patients with fresh, uncomplicated and closed fractures of the facial skeleton.

Antibiotics differ significantly, as far as their prices are concerned. Heit *et al.*^18^ compared the costs of 1-day therapy with cetrifaxone and penicillin G that were used for the prophylaxis of surgical treatment of mandibular fractures. They proved that cetrifaxone is more efficacious and ~$350 (1,400 PLN) less expensive than penicillin G. The costs of 1 day’s therapy with antibiotics or chemotherapeutic agents to which Gram-positive bacteria had similar sensitivity (>80%) was calculated with the drugs: trimethoprim/sulfamethoxazole, imipenem, ciprofloxacin and gentamycin. Ciprofloxacin was the least expensive antibiotic with efficacy against Gram-positive bacteria of 80% and >89% against Gram-negative bacteria (the cost of 1-day therapy is ~2 PLN). The most expensive antibiotic is imipenem (~300 PLN). The most efficacious drug here is gentamycin, in which case the daily cost of treatment is ~3 PLN. Gram-negative bacteria display a similar sensitivity to imipenem, ciprofloxacin and gentamycin. The cost for the treatment of methicyllin-resistant *Staphylococcus aureus* (MRSA) skin and soft tissue is the lowest for linezolid, whereas the total cost of hospital treatment of such infections with antibiotics is the lowest for vancomycin. Our studies confirm these observations for infections caused by *Str. mitis* and *Str. oralis*, where vancomycin was the most efficacious from the perspective of pharmacoeconomics.^3^ The difference in the bacterial sensitivity makes it a challenge to create pharmocoeconomical analysis that could apply to other countries. Poveda Roda *et al.* by reviewing the previous study references concluded that amoxicillin with clavulanic acid, moxifloxacin and clindamycin are preferred for bacterial infection of dental origin. However, for nondental origin infections, it is recommended to use clindamycin and fluoroquinolones, preferably moxifloxacin, in order to cover the spectrum including anaerobic bacteria. Given our results from the past 2 years concerning the bacterial sensitivity to clindamycin and amoxicillin+clavulanic acid, being 60.2% and 59.8% respectively, this therapy will not be efficacious.^19^

Kuriyama *et al.*^20^ have observed lower bacterial resistance to penicillin (38%), as compared to the 49.4% being the result of our study. However, they did not find any statistical differences in the therapeutic effect on using various antibiotics in alveolar osteitis post extraction.

In the past 2 years, *Streptococcus* was the most frequently isolated bacterial strain.

The antibiotics that are the most efficacious against *Str. mitis* and *Str. oralis* are penicillin and ampicillin. From the point of view of pharmacoeconomics, gentamycin is the most advantageous antibacterial agent, effective against *S. epidermidis*, whereas vancomycin turns out to be the most efficacious against *Str. mitis* and *Str. oralis*. In the case of *S. aureus*, the best antibiotics are gentamycin and trimethoprim/sulfamethoxazole. The resistance results suggest that empirical therapy should be based on ciprofloxacin and gentamycin.

## Disclaimer

This research did not receive any specific grant from funding agencies in the public, commercial or not-for-profit sectors.

## Figures and Tables

**Table 1:** Characterisation of bacteria isolates

*Number of pathogens*	*Number of patients*	*Total number of cultured strains*
1	111	111
2	101	202
3	32	96
4	19	76
Total	263	485

**Table 2:** Antibiotic sensitivity of *S. aureus*, *S. epidermidis*, *Str. mitis* and *Str. oralis*

	*Trimethoprim/sulfamethoxazole*	*Vancomycin*	*Clindamycin*	*Gentamycin*	*Tetracyclin*	*Erythromycin*	*Teicoplanin*	*Ampicillin*	*Penicillin G*	*Ceftriaxone*
S. epidermidis *88 strains (19%)*										
Drug sensitivity										
Number	81	88	49	80	42	46	NA	NA	NA	NA
%	92	100	56	91	48	52	NA	NA	NA	NA
Drug resistance										
Number	3	0	19	2	23	42	NA	NA	NA	NA
%	8	0	44	9	52	48	NA	NA	NA	NA
										
Str. mitis *and* Str. orali*s 67 strains (13.8%)*										
Drug sensitivity										
Number	NA	66	40	NA	NA	NA	NA	35	48	40
%	NA	99	60	NA	NA	NA	NA	52	72	60
Drug resistance										
Number	NA	1	27	NA	NA	NA	NA	32	19	27
%	NA	1	40	NA	NA	NA	NA	48	28	40
										
S. aureus *31 strains (6.4%)*										
Drug sensitivity										
Number	29	31	27	29	23	26	31	NA	NA	NA
%	94	100	87	94	74	84	100	NA	NA	NA
Drug resistance										
Number	2	0	4	2	8	5	0	NA	NA	NA
%	6	0	13	6	26	16	0	NA	NA	NA
										
*Treatment cost (PLN)*										
Route of administration	i.v.	i.v.	i.v.	i.v.	p.o.	p.o.	i.v.	i.v.	i.v.	i.v.
1 day	8.3	31.4	23.26	3.02	6.32	5.96	172.37	12.12	13.42	13.42
7 days	58.1	219.8	162.82	21.14	44.24	41.72	1206.59	84.84	93.94	93.94
10 days	83	314	232.6	30.2	63.2	59.6	1723.7	121.2	134.2	134.2
14 days	116.2	439.6	325.64	42.28	88.48	83.44	2413.18	169.68	187.88	187.88

Abbreviations: i.v., intravenous; NA, not available; p.o, per os. oral administration.
